# FOOTBIKE exercise enhances mental health through improved executive function in university students

**DOI:** 10.3389/fpsyg.2026.1891734

**Published:** 2026-10-26

**Authors:** Shuo Qi, Jinglun Yu, Zhen Wei, Lijun Yin, Lai Wei

**Affiliations:** 1 School of Sport and Health, Shandong Sport University, Jinan, China; 2 School of Sport and Health Science, Xi'an Physical Education University, Xi'an, China; 3 School of Exercise and Health, Nanjing Sport Institute, Nanjing, China; 4 School of Sport, Shenzhen University, Shenzhen, China; 5 Shanghai Investigation, Design & Research Institute, Shanghai, China

**Keywords:** executive function, FOOTBIKE, mental health, university sports, university students' mental health

## Abstract

**Introduction:**

This study investigated the effects of FOOTBIKE exercise on mental health and executive function in university students, aiming to explore a novel exercise-based approach for mental health promotion in higher education settings.

**Methods:**

Sixty healthy undergraduate students from Shandong Sport University were randomly assigned to either an experimental group (*n* = 30) or a control group (*i* = 30). The experimental group completed an 8-week FOOTBIKE intervention consisting of three 60-min sessions per week, while the control group maintained routine activities and regular physical education classes. Pre- and post-intervention assessments utilized an AI-based physiological and psychological monitoring system, a 2-back working memory task, and a Go/No-Go inhibitory control task.

**Results:**

Following the FOOTBIKE exercise intervention, the experimental group exhibited significantly reduced anxiety and inhibition indices compared to the control group (*p* < 0.05), together with significantly enhancing reaction capacity and stress resilience (*p* < 0.05). Reaction times in both the 2-back working memory task and the Go/No-Go inhibitory control task were also significantly reduced after FOOTBIKE training (*p* < 0.05), indicating improvements in cognitive processing efficiency and inhibitory control. There was a significant group 7 time interaction effect on the inhibition index, reaction times in the 2-back task, and reaction times in the Go/No-Go task. In contrast, no significant differences were observed in conventional physiological indicators or task accuracy, suggesting that the psychological benefits of FOOTBIKE exercise may be more closely related to cognitive and emotional regulatory adaptations than to general physiological conditioning alone. Furthermore, random forest analysis identified reaction speed, anxiety index, stress tolerance, and executive-function-related variables as important contributors distinguishing the experimental group after intervention, which was further supported by principal component analysis.

**Conclusion:**

FOOTBIKE exercise enhances mental health primarily through improvements in executive function, emotional regulation, cognitive responsiveness, and stress adaptation capacity. As a cognitively engaging, enjoyable, safe, and easily scalable form of physical activity, FOOTBIKE may represent a promising intervention strategy for university physical education curricula and mental health promotion programs.

## 1 Introduction

University students are at a critical stage of psychological and social development, during which they are frequently exposed to multiple pressures related to academic demands, career prospects, and interpersonal relationships (Guo, 2025). Consequently, mental health problems among university students have become increasingly prevalent and diversified in recent years. Previous studies have reported high rates of anxiety, depression, emotional instability, attentional difficulties, and insufficient stress-coping abilities within this population (Ramón-Arbués et al., 2020; Vicent et al., 2025; Woodson et al., 2025). In this context, mental health—the capacity to adapt positively to adversity, regulate emotions effectively, and maintain cognitive and behavioral stability under stress—has emerged as an important protective factor for psychological wellbeing. Importantly, mental health is increasingly recognized not only as an emotional trait, but also as a multidimensional construct closely associated with executive function, cognitive control, stress adaptation, and self-regulation ability (Rutten et al., 2013). Traditional university mental health education primarily relies on classroom-based psychological instruction, counseling services, and thematic educational activities. Although these approaches play an important role in mental health support, they often face limitations such as low participation rates, insufficient experiential engagement, and potential psychological stigma. In recent years, exercise-based interventions have attracted growing attention in mental health promotion research because of their non-stigmatizing nature, high accessibility, group interaction characteristics, and positive emotional effects (Donnelly et al., 2024; Solmi et al., 2025). Studies demonstrate that regular physical activity, particularly moderate-intensity aerobic exercise, can improve emotional wellbeing, reduce anxiety and stress, and enhance executive function and mental health (Donnelly et al., 2024; Smith and Merwin, 2021). However, the psychological benefits of exercise may not depend solely on physiological conditioning, but also on the degree of cognitive engagement involved in movement participation. Exercise modalities requiring simultaneous motor coordination, attentional regulation, inhibitory control, and environmental interaction may produce stronger benefits for executive function and mental health than repetitive physical activity alone. Therefore, further exploration of cognitively engaging exercise modalities is warranted.

Executive function, including working memory, inhibitory control, and attentional regulation, plays a central role in emotional regulation and adaptive coping under stressful conditions. According to Diamond (2013), executive function is fundamental to self-regulation, goal-directed behavior, and flexible adaptation to environmental challenges (Diamond, 2013). Individuals with stronger executive function are generally better able to regulate emotional responses, suppress maladaptive behaviors, and maintain cognitive stability during stressful situations. Previous studies have demonstrated that physical activity can enhance executive function through increased neuroplasticity and activation of prefrontal cognitive control systems (Zhang et al., 2025). Consequently, exercise interventions capable of simultaneously stimulating motor coordination and cognitive control may represent a promising strategy for improving mental health in university students.

FOOTBIKE is an emerging full-body coordination activity that combines elements of scooting, running, and cycling into a rhythmic whole-body coordination activity. Movement is achieved through repeated gliding, pedaling, and coasting actions (Figure 1). Compared with traditional repetitive aerobic exercise, FOOTBIKE requires participants to continuously regulate movement rhythm, posture, balance, route judgment, and environmental perception, thereby imposing sustained demands on cognitive control and motor coordination systems. This characteristic aligns with the concept of “cognitively engaging physical activity,” which emphasizes the integration of cognitive processing and motor execution during exercise participation. In addition, FOOTBIKE possesses several features potentially beneficial for mental health promotion, including moderate exercise intensity, strong enjoyment, high accessibility, low impact, and flexible adaptability to different exercise environments. The speed experience, rhythmic movement patterns, outdoor environmental exposure, and social interaction involved in FOOTBIKE activities may collectively contribute to positive emotional activation, attentional regulation, stress reduction, and adaptive coping enhancement.

**Figure 1:**
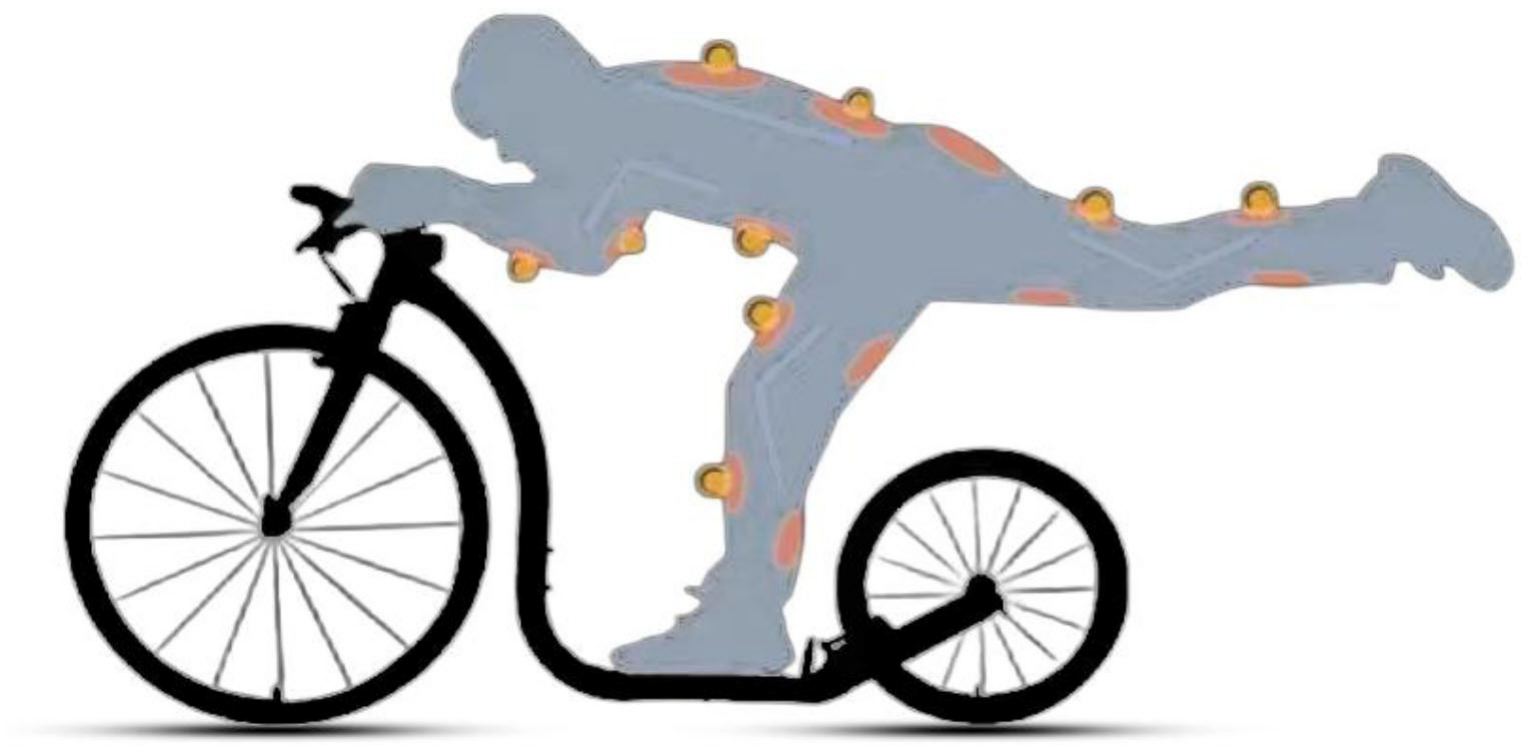
FOOTBIKE gliding vehicle sport.

Although previous studies have suggested that gliding or rhythm-based exercise modalities may improve attention control, emotional regulation, and cognitive performance (Dkaidek et al., 2023), research specifically examining the effects of FOOTBIKE exercise on mental health and executive function in university students remains extremely limited. In particular, the potential mechanisms linking cognitively engaging exercise, executive function enhancement, and mental health have not yet been fully clarified.

Therefore, the present study employed a randomized controlled trial design to investigate the effects of an 8-week FOOTBIKE exercise intervention on mental health, emotional regulation, executive function, and stress adaptation in university students. By integrating psychological assessment, executive-function tasks, and machine learning analyses, this study aimed to explore whether FOOTBIKE exercise could promote mental health through improvements in cognitive control and executive functioning. The findings may provide new theoretical perspectives and practical approaches for exercise-based mental health promotion in university settings.

## 2 Methods

### 2.1 Participants

This study selected healthy undergraduate students from Shandong Sport University as experimental subjects. An a priori sample size calculation was performed using G^*^Power software (Version 3.1.9.2; Heinrich Heine University Düsseldorf, Germany). The required sample size was estimated for a 2 (group: experimental vs. control) × 2 (time: pre- vs. post-intervention) mixed-design repeated-measures analysis of variance (ANOVA) using the “ANOVA: Repeated measures, within–between interaction” procedure. Based on a medium effect size (Cohen's f = 0.25), a significance level of α = 0.05, a desired statistical power of 0.80, two groups, two repeated measurements, an assumed correlation of 0.50 between repeated measurements, and a no sphericity correction of 1.0, the analysis indicated that a minimum total sample size of 34 participants was required. To account for potential attrition, a total of 60 healthy undergraduate students were recruited as participants (Table 1). All subjects were in good physical condition, free from chronic diseases or severe medical complications, without movement disorders or exercise contraindications, had no prior fitness experience, no joint or ligament injuries, and were not taking nutritional supplements or medications. This study was approved by the Ethics Review Committee of Shandong Sport University (Approval No.: 2025072). Prior to the experiment, all subjects were informed of the experimental procedures and precautions and signed informed consent forms. Within 24 h before each experiment, subjects ensured adequate sleep, avoided alcohol, caffeine, and nicotine intake, and refrained from strenuous exercise.

**Table 1:** Basic information of participants.

Group	Age	Height (cm)	Weight (kg)	*N*
Experimental Group	18 ± 0.5	173.2 ± 6.2	63.4 ± 10.5	30
Control Group	18 ± 0.8	173.6 ± 6.6	63.6 ± 11.1	30

### 2.2 FOOTBIKE exercise intervention protocol

The 8-week FOOTBIKE intervention was structured around clearly defined weekly objectives and corresponding training content. The objectives specified the skills or capacities to be developed at each stage, whereas the training content provided the specific activities and progression used to achieve these objectives. The program progressed from basic gliding control and rhythm establishment to endurance development, speed and endurance control, power generation, uphill and gear-shifting skills, sustained gliding, and finally posture adjustment, behavioral reinforcement, and timed/team challenges. Training distance progressively increased across the first seven weeks, while the frequency and duration remained constant at three sessions per week and 60 min per session. The weekly progression was therefore primarily determined by the predefined intervention objectives and the need to gradually increase skill and exercise demands, rather than by unsystematic changes based on individual participant needs. Although participants were supervised throughout the intervention, no formal individualized adjustment protocol was implemented. This objective-oriented and content-matched structure was intended to ensure that each stage provided appropriate activities for achieving its corresponding training objective while maintaining a consistent and progressive intervention framework.

The FOOTBIKE exercise intervention protocol is outlined in Table 2. The experimental group underwent an 8-week continuous intervention, three times a week. The FOOTIKE exercise sessions were scheduled for Mondays, Wednesdays and Fridays from 3.00 p.m. to 4.00 p.m., with each session lasting 60 min.

**Table 2:** FOOTBIKE gliding vehicle exercise program.

Week	Training objective	Training content	Ski distance	Schedule
Week 1	Basic gliding control	Practice mounting, grip width, pushing off, and braking; single-leg gliding and foot-switching exercises	3 km	3 times per week, 60 min per session
Week 2	Establish gliding rhythm and symmetrical power generation on both sides	Continuous cross-over gliding, smooth gliding at medium to low speeds	4 km	3 times per week, 60 min per session
Week 3	Improve Cardiorespiratory Endurance	Perform three cycles of continuous gliding at moderate to low speeds for 20–25 min each.	5 km	3 times per week, 60 min per session
Week 4	Improve speed and endurance control	Interval training: Cycle through 5 minutes of moderate-speed skating and 2 mins of faster skating multiple times.	6 km	3 times per week, 60 min per session
Week 5	Optimize pedaling power generation and gliding efficiency	Enhanced hip-driven power generation in the back kick	7 km	3 times per week, 60 min per session
Week 6	Mastering uphill gliding and gear shifting techniques	Practice gliding uphill on small slopes and adjusting speed	8 km	3 times per week, 60 min per session
Week 7	Enhance sustained gliding ability	Maintain a steady glide at moderate speed for 30–40 min to establish a consistent gliding rhythm	9 km	3 times per week, 60 min per session
Week 8	Adjust gliding posture and reinforce behavioral habits	Participate in 5–10 km timed gliding challenges and team relay events	5 km Timed Glide Challenge	3 times per week, 60 min per session

Participants assigned to the control group maintained their regular university lifestyle throughout the 8-week study period and did not receive any FOOTBIKE intervention or additional structured exercise training. Their daily routine consisted of attending scheduled university courses (including College Chinese and Applied Writing, Sports Anatomy, Pedagogy, College Physical Education, College Foreign Language, Introduction to Sports, Fundamentals of Traditional Chinese Medicine, Ideology, Morality and Rule of Law, and Fundamentals of Innovation and Entrepreneurship), self-study, meals, and routine daily activities. Apart from the compulsory physical education classes required by the university curriculum, participants were instructed not to engage in any additional organized exercise or sports training during the intervention period. The daily routine of the control group of college students can be found in the Supplementary material.

### 2.3 Experimental testing metrics

Participants underwent two tests: one prior to the intervention (baseline) and the other immediately following the 8-week intervention (post-intervention). All tests were conducted between 08:00 and 11:30 in the laboratory at Shandong University of Physical Education to minimize the effects of circadian rhythm variations. Participants were instructed to avoid strenuous physical activity, caffeine and alcohol for 24 h prior to the tests. Upon arrival at the testing venue, participants first underwent the AI-Psychophysiological Health Assessment System test, followed by cognitive function tests (2-back/Go/No-Go). A standardized 5-min rest period was provided between consecutive tests to minimize fatigue. All assessments were conducted by trained researchers using standardized protocols under identical environmental conditions.

The AI-Psychological and Physiological Health Monitoring System was used to assess multiple physiological and psychological health indicators in subjects (Figure 2). All participants underwent baseline testing prior to the experiment and post-test afterward, with consistent standards and methodologies applied for each assessment. The AI-based physiological and psychological assessment system evaluated three categories of indicators. Physiological health indicators included heart rate, blood oxygen saturation, heart rate variability, respiratory rate, systolic blood pressure, and diastolic blood pressure. Emotional indicators included the irritability index, stress index, anxiety index, self-confidence index, vitality index, inhibition index, neuroticism index, depression index, pleasure index, composure, personality orientation, and self-regulation. Behavioral and adaptive functioning indicators included communication and expression skills, reaction speed, stress tolerance, learning adaptability, self-management skills, risk tolerance, innovation capabilities, proactive execution, and crisis management skills.

**Figure 2:**
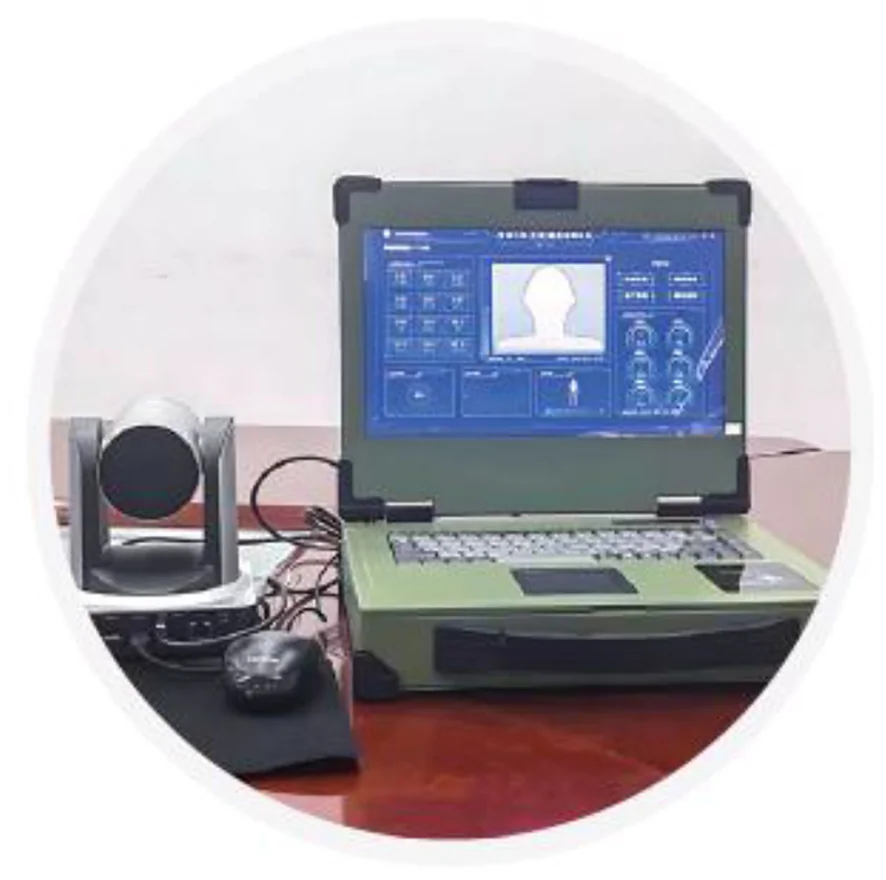
AI-based mental and physical health monitoring system (image reproduced with permission from the 'AI Mental and Physiological Health Assessment System Manual', Today Education Group's Shixinzhe Technology [Nanjing] Co., Ltd).

Technical Principle 1 (Figure 3): Optical Imaging Technology for Blood Flow Information. Human skin reflects incident light, and periodic changes in subcutaneous blood volume produce subtle variations in facial skin color that can be captured using digital cameras. These color fluctuations form the basis of remote photo plethysmography, enabling non-contact extraction of physiological information such as blood flow and heart rate (Allen, 2007; Poh et al., 2010; Verkruysse et al., 2008). The recorded facial videos are processed using temporal and frequency-domain filtering techniques based on the Eulerian Video Magnification framework to amplify subtle color variations that are imperceptible to the naked eye, thereby reconstructing physiological signals from facial blood flow changes (Wu et al., 2018). Subsequently, the extracted physiological features are input into a deep neural network for automated estimation of multidimensional physiological and psychological indicators.

**Figure 3:**
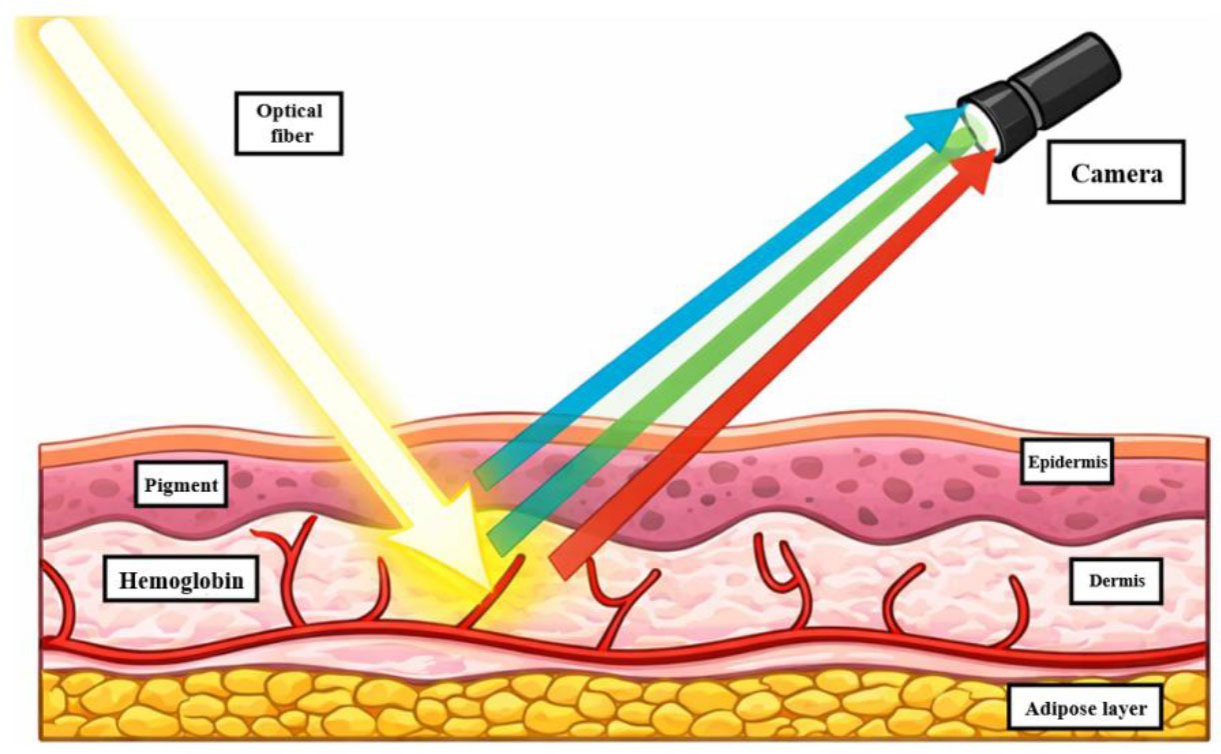
Blood flow information optical imaging technology (image reproduced with permission from the 'AI Mental and Physiological Health Assessment System Manual', Today Education Group's Shixinzhe Technology [Nanjing] Co., Ltd).

Technical Principle 2 (Figure 4): Video Image Micro-Vibration Analysis Technology employs high-frame-rate cameras to rapidly capture images of minute muscle movements in the head, neck, and face regions of the subject. Combined with computer analysis, these images are converted into pixel-level motion data, capturing three-dimensional movement trajectories. The amplitude and frequency of vibration at each pixel point during motion are then calculated. By correlating the cumulative changes in pixel positions across time-differenced frames and comparing them against the system's fundamental feature model database, the technology analyzes the subject's potential emotional states and various psychological indicator parameters. Muscle micro-movements under autonomic nervous system influence constitute unconscious, physiologically objective reactions beyond conscious control. Because facial micro-movements are influenced by autonomic nervous system activity and are less susceptible to voluntary control than self-reported responses, video-based facial motion analysis provides an objective source of physiological information that may complement conventional psychological assessment methods (Kreibig, 2010). Consequently, psychological and emotional indicators derived through this technology exhibit fully objective authenticity and reliable, non-deceivable characteristics.

**Figure 4:**
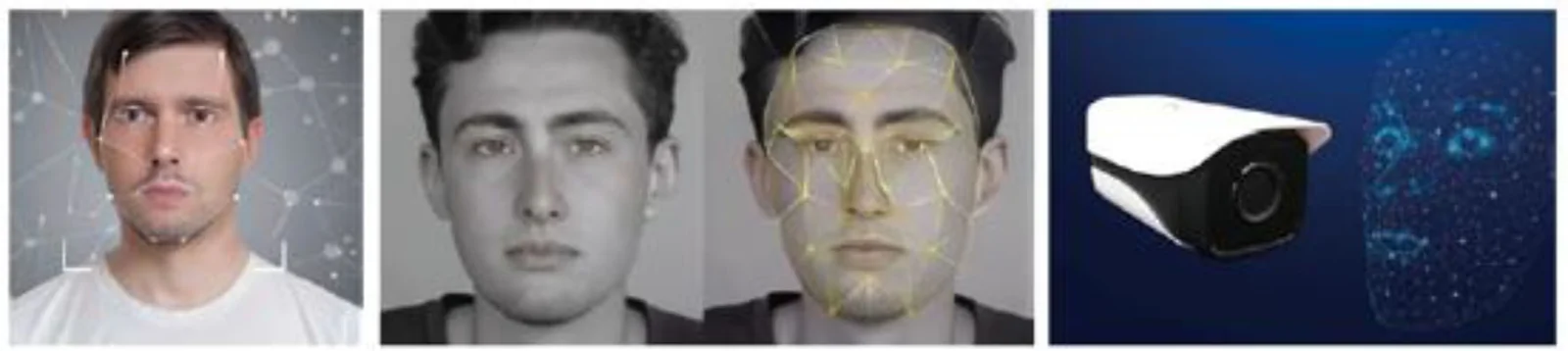
Video image micro-vibration analysis technology (image reproduced with permission from the 'AI Mental and Physiological Health Assessment System Manual', Today Education Group's Shixinzhe Technology [Nanjing] Co., Ltd).

#### 2.3.2 Working memory measurement

A 2-back task was employed, consisting of 4 blocks, each containing 15 stimuli. Initially, a fixation point appeared on the computer screen for 500 ms, followed by the random presentation of one of the 8 English letters: B, D, H, K, M, P, S, or Y. Each letter was displayed for 1000 ms, with 2000 ms intervals between letters. After the participant responds, the next stimulus appears. Stimuli exceeding the response time are recorded as “no response.” Participants must begin memorizing upon the third letter's appearance, determining whether the current letter matches the letter preceding the previous one. Press ‘F' for a match and “J” for a mismatch. The formal experiment begins once the participant achieves an 85% accuracy rate during practice.

#### 2.3.2 Inhibition control measurement

Employed a Go/Nogo task divided into 4 blocks, with a 15-second rest between each block. Task stimuli consisted of black “W” and “M” letters. The experiment included both practice and formal phases, each containing both Go and Nogo stimuli. The ratio of Go to Nogo stimuli was 3:1. The formal experiment comprised 80 stimuli, with each block containing 16 Go stimuli (80%) and 4 Nogo stimuli (20%). For each stimulus, a black “W” (Go stimulus) or ‘M' (Nogo stimulus) appeared randomly in the center of the screen. For “W,” participants pressed the spacebar as quickly as possible while ensuring accuracy. For “M” (Nogo stimulus), participants refrained from any response. Participants practiced under the experimenter's guidance until their practice accuracy exceeded 90%, after which they proceeded to the formal test.

### 2.4 Statistical analysis

Experimental data were processed using SPSS 26.0 software. All data are presented as mean ± standard deviation (M ± SD). A paired *t-test* was used to compare the pre- and post-experimental data within each group, whilst repeated measures analysis of variance (ANOVA) was used to assess differences between groups. A *p*-value of < 0.05 was defined as statistically significant. When the results of the repeated measures ANOVA revealed a time × group interaction effect, a simple effects analysis was conducted. The random forest (RF) model, a machine learning method, was used to identify the key indicators for each group, and principal component analysis (PCA) was employed to differentiate between the groups.

## 3 Results

As shown in Figure 5 and Table 3, no significant between-group differences were observed in physiological indicators, including heart rate, blood oxygen saturation, heart rate variability, respiratory rate, or systolic blood pressure following the 8-week intervention (*p* > 0.05). These findings suggest that the beneficial effects of FOOTBIKE exercise on mental health were not primarily driven by changes in basic physiological status.

**Figure 5:**
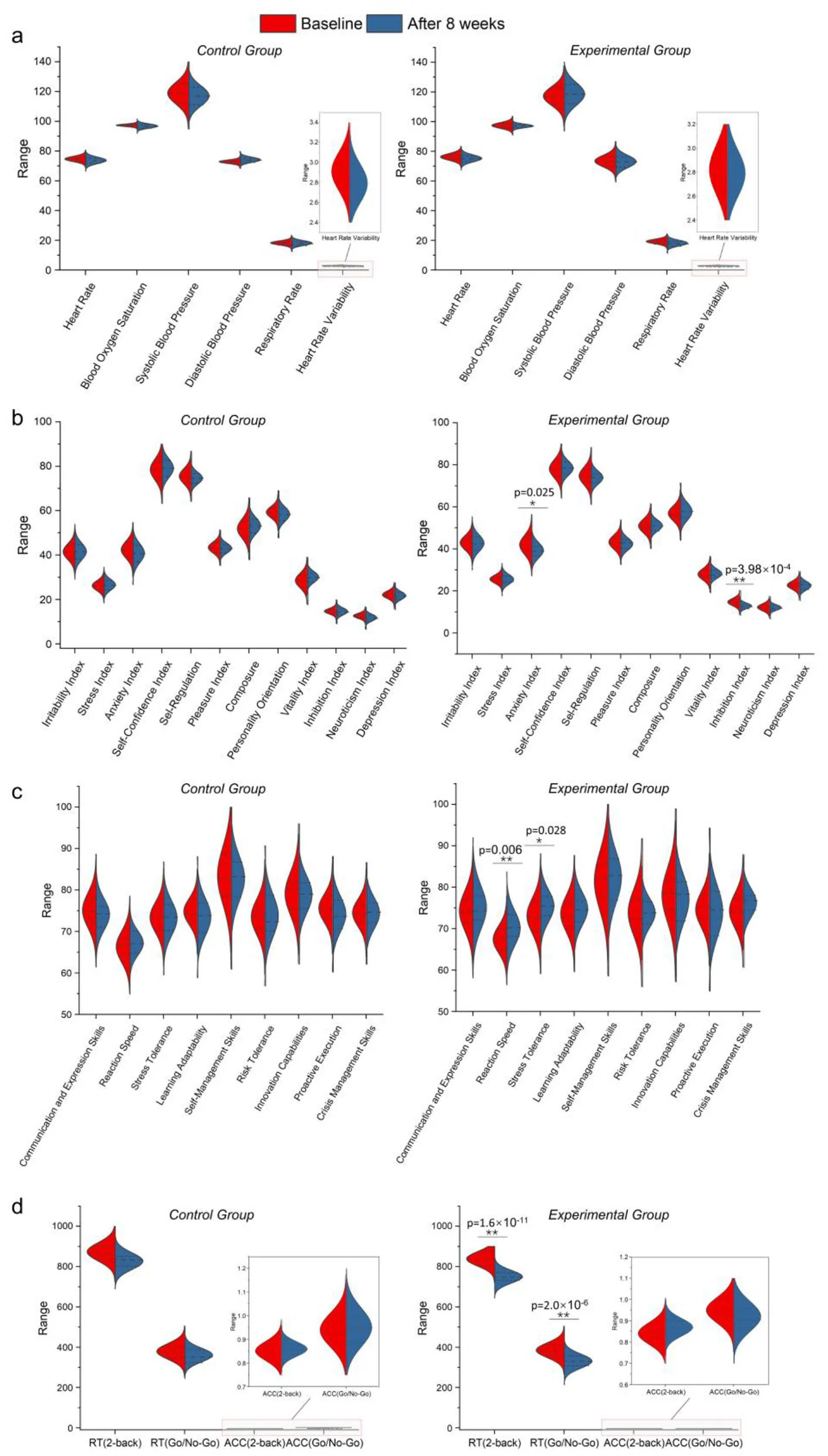
Half-violin plots comparing baseline and 8-week measurements between the control and experimental groups across four dimensions: physiological health **(a)**, mental health **(b)**, competency **(c)**, and executive function **(d)**. Each group included 30 participants (control group: *n* = 30; experimental group: (*n* = 30), and all participants were assessed at baseline and after 8 weeks. Half-violin plots show the distribution of individual values, with boxplots indicating the median and interquartile range (IQR). Significance levels are denoted as ** (*p* < 0.01) and * (*p* < 0.05) with exact *p*-values.

**Table 3:** The effects of FOOTBIKE gliding exercise on four dimensions among university students.

Dimensions	Indicators	Control group—baseline	Control group-−8 weeks	Experimental group—baseline	Experimental group-−8 weeks
Physiological health	Heart rate	74.9 ± 2.8	73.7 ± 2.1	76.2 ± 2.2	75.2 ± 2.3
	Blood oxygen saturation	97.3 ± 1.1	96.8 ± 1.4	97.4 ± 1.9	97.1 ± 1.2
	Heart rate variability	2.91 ± 0.19	2.79 ± 0.19	2.82 ± 0.21	2.79 ± 0.19
	Respiratory rate	18.2 ± 1.3	18.1 ± 1.6	19.2 ± 1.5	18.1 ± 1.9
	Systolic blood pressure	118.9 ± 7.2	117.1 ± 6.7	117.2 ± 6.2	118.6 ± 7.5
	Diastolic blood pressure	72.8 ± 1.1	74.1 ± 1.7	73.4 ± 3.8	73.1 ± 4.1
Emotional	Irritability index	41.5 ± 3.5	41.6 ± 3.7	42.9 ± 3.3	42.7 ± 3.8
	Stress	26.2 ± 2.2	26.6 ± 2.4	25.6 ± 1.9	25.5 ± 2.3
	Anxiety	42.4 ± 3.7	40.8 ± 4.2	41.9 ± 4.4	39.3 ± 3.6^*^
	Self-confidence index	78.5 ± 4.6	79.1 ± 4.2	78.1 ± 4.2	78.4 ± 3.4
	Vitality index	28.5 ± 3.2	30.1 ± 2.4	27.9 ± 2.6	28.2 ± 2.2
	Inhibition index	14.7 ± 1.2	14.6 ± 1.6	14.7 ± 1.7	12.9 ± 1.3^**^
	Neuroticism index	12.6 ± 1.1	11.8 ± 1.5	12.2 ± 1.6	12.1 ± 1.3
	Depression index	22.1 ± 1.6	21.7 ± 1.8	22.7 ± 1.9	22.7 ± 2.1
	Pleasure index	43.4 ± 2.4	43.1 ± 2.2	43.2 ± 3.2	42.3 ± 2.9
	Composure	51.9 ± 4.2	53.2 ± 3.6	50.9 ± 3.2	51.1 ± 2.6
	Personality orientation	59.3 ± 2.5	58.4 ± 3.2	56.9 ± 3.4	57.8 ± 4.1
	Self-regulation	75.5 ± 3.4	74.6 ± 2.6	74.7 ± 4.1	73.9 ± 3.1
Behavioral and adaptive functioning	Communication and expression skills	75.1 ± 4.1	74.1 ± 3.7	74.1 ± 4.8	75.1 ± 5.1
	Reaction speed	66.3 ± 3.7	67.2 ± 2.9	67.4 ± 3.2	70.1 ± 4.1^**^
	Stress Tolerance	73.4 ± 3.6	73.2 ± 4.1	73.1 ± 4.2	75.5 ± 3.8^*^
	Learning adaptability	74.8 ± 3.1	73.5 ± 4.4	73.6 ± 4.2	74.8 ± 3.9
	Self-management skills	83.5 ± 6.8	82.8 ± 5.4	81.5 ± 6.9	82.4 ± 6.1
	Risk tolerance	73.5 ± 4.2	73.8 ± 5.1	73.9 ± 5.4	73.8 ± 3.4
	Innovation capabilities	79.1 ± 5.1	78.9 ± 4.4	78.1 ± 6.3	77.1 ± 5.7
	Proactive execution	75.6 ± 3.3	74.2 ± 4.2	74.6 ± 4.1	74.1 ± 6.1
	Crisis management skills	75.6 ± 3.2	74.4 ± 3.7	74.3 ± 4.1	76.2 ± 3.4
Executive function	RT (2-back)	873.13 ± 45.2	826.89 ± 41.2	838.28 ± 35.6	749.53 ± 31.5^**^
	ACC (2-back)	0.85 ± 0.04	0.86 ± 0.03	0.84 ± 0.05	0.87 ± 0.04
	RT (Go/No-Go)	379.06 ± 38.4	361.05 ± 34.1	384.91 ± 36.4	330.89 ± 34.5^**^
	ACC (Go/No-Go)	0.94 ± 0.07	0.95 ± 0.08	0.95 ± 0.06	0.92 ± 0.07

^*^p < 0.05, ^**^p < 0.01, compared with the experimental group baseline.

In contrast, significant improvements were observed in several psychological and executive-function-related indicators in the experimental group. Specifically, the experimental group demonstrated significantly lower anxiety index and inhibition index values compared with the control group (*p* < 0.05), indicating enhanced emotional regulation and inhibitory control after the FOOTBIKE intervention. However, no significant differences were found in irritability index, stress index, self-confidence index, or vitality index (*p* > 0.05).

Regarding competency-related indicators, the experimental group exhibited significant improvements in reaction ability and stress resistance (*p* < 0.05), whereas no significant changes were observed in communication skills, learning adaptability, self-management ability, or risk resistance (*p* > 0.05). These findings indicate that FOOTBIKE exercise may preferentially enhance cognitive responsiveness and stress adaptation rather than broadly improving all competency dimensions.

Consistent with these findings, performance in executive function tasks also improved significantly after the intervention. The experimental group showed significantly reduced reaction times (RTs) in both the 2-back working memory task and the Go/No-Go inhibitory control task (*p* < 0.05), while no significant differences were observed in task accuracy (ACC) (*p* > 0.05). The reduction in RT without a decline in ACC suggests that FOOTBIKE exercise enhanced cognitive processing efficiency and inhibitory control rather than inducing a speed–accuracy trade-off.

To further evaluate the multidimensional effects of FOOTBIKE exercise, a random forest (RF) model was constructed using psychological, cognitive, and selected physiological indicators. Given the high inter-correlations among physiological variables (Supplementary Figure S4), only blood oxygen saturation was retained as a representative physiological input variable (Supplementary material). The RF model was evaluated using repeated 10-fold cross-validation and achieved the highest mean accuracy (76.7%) and Kappa value (0.689) when mtry = 2, indicating acceptable classification performance. Feature importance analysis further demonstrated that several indicators associated with executive function and mental health showed higher contributions in the experimental group after 8 weeks, particularly reaction speed, anxiety index, stress tolerance, inhibition index, and RT in the 2-back task (Figure 6). These findings suggest that FOOTBIKE exercise may improve mental health primarily through enhanced cognitive control and stress adaptation mechanisms.

**Figure 6:**
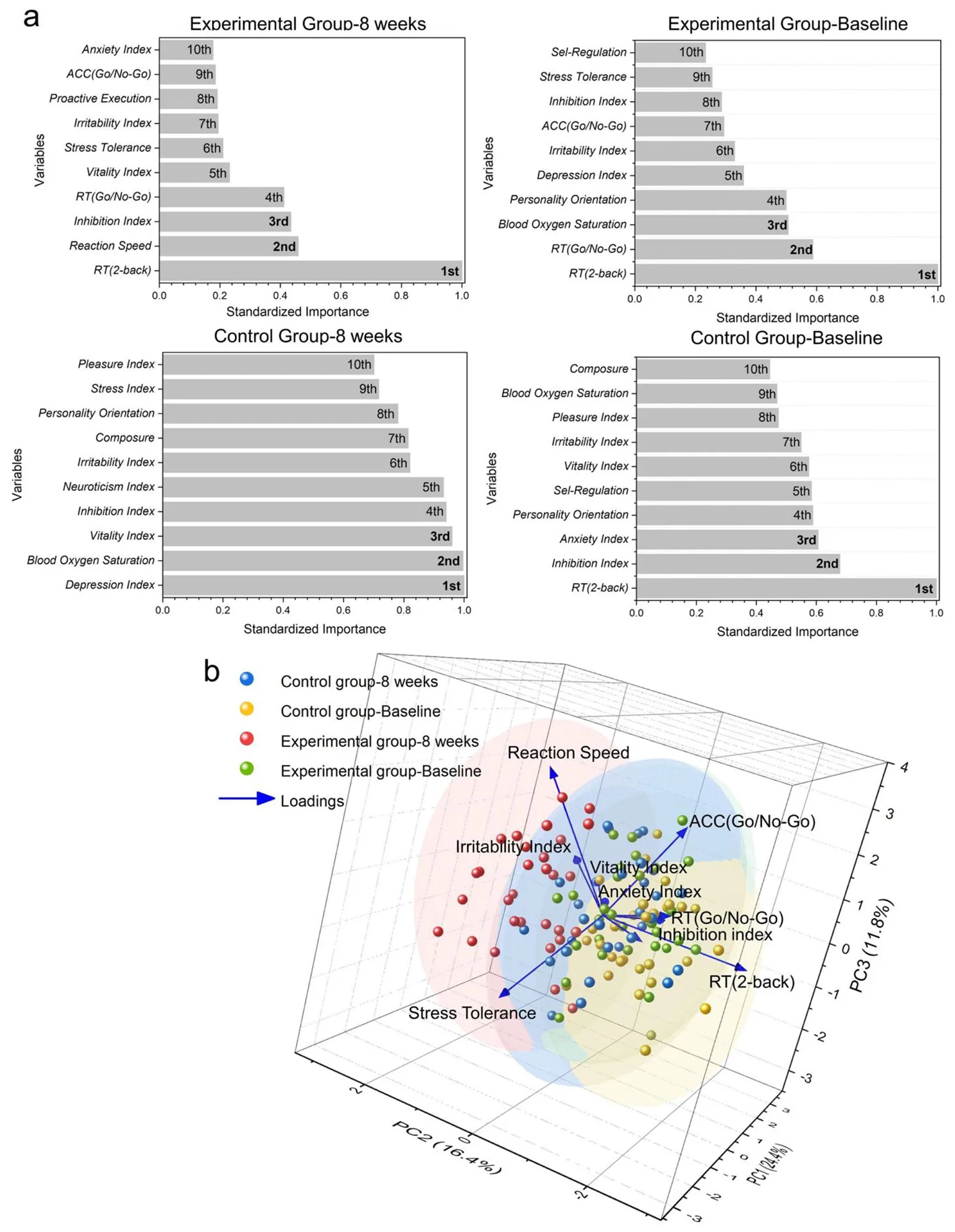
Importance ranking (top ten) for each group based on the RF model **(a)**, and PCA plots derived from the key indicators **(b)**. In (b), the percentages shown on the axes represent the variance explained by PC1 and PC2, and the ellipses indicate the 95% confidence regions for each group. Each group comprised 30 participants (*n* = 30), who were assessed at baseline and after 8 weeks.

The RF results were further supported by principal component analysis (PCA). As shown in Figure 6, the experimental group after 8 weeks was clearly separated from the other groups in multidimensional feature space, mainly driven by variables related to reaction speed, stress tolerance, inhibitory control, and executive task performance. Collectively, these findings indicate that FOOTBIKE exercise induces coordinated improvements in executive function and mental health in university students.

This study employed a two-way mixed-design repeated-measures analysis of variance to examine the effects of group (experimental group and control group) and time (pre-intervention and post-intervention) on physiological, psychological and cognitive performance indicators. As shown in Table 4, the results of the repeated measures analysis of variance revealed a significant group × time interaction for the inhibition index, RT (2-back) and RT (Go/No-Go), indicating that there were significant differences in the changes over time between the experimental and control groups (*P* < 0.05). With regard to the inhibition index, significant main effects of time and group were also observed, indicating overall differences between assessment time points and between groups (*P* < 0.01). Similarly, RT (2-back) showed significant main effects of time and group (*P* < 0.01), indicating an overall reduction in reaction time following the intervention, with the experimental group performing superiorly. For RT (Go/No-Go), a significant main effect of time was detected (*P* < 0.01), whilst the main effect of group did not reach statistical significance. Among the remaining variables, time had significant main effects on heart rate variability, anxiety index, vitality index and reaction speed, whilst group had significant main effects on heart rate, vitality index and reaction speed (*P* < 0.05). No significant group × time interactions were found for these measures, indicating that the temporal changes across groups were comparable. In view of the significant interaction effects observed for the inhibition index, RT (2-back) and RT (Go/No-Go), *post-hoc* simple effects analyses were subsequently conducted to further characterize the differences in changes over time between groups.

**Table 4:** Repeated measures ANOVA of various indicators among college students before and after the experiment.

Indicators	Effect	Class III sum of squares	Degree of freedom	Mean square	F	*P*
Heart rate	Time	28.587	1	28.587	6.071	0.017
	Group	69.662	1	69.662	15.979	0.000^**^
	Time^*^Group	0.017	1	0.017	0.004	0.952
Blood oxygen Saturation	Time	5.002	1	5.002	2.459	0.122
	Group	1.102	1	1.102	0.585	0.448
	Time^*^Group	0.352	1	0.352	0.173	0.679
Heart rate variability	Time	0.169	1	0.169	4.400	0.040^*^
	Group	0.061	1	0.061	1.505	0.225
	Time^*^Group	0.061	1	0.061	1.583	0.213
Respiratory rate	Time	10.8	1	10.8	3.886	0.054
	Group	7.5	1	7.5	3.074	0.085
	Time^*^Group	7.5	1	7.5	2.685	0.107
Systolic blood pressure	Time	1.2	1	1.2	0.022	0.882
	Group	0.3	1	0.3	0.007	0.935
	Time^*^Group	76.8	1	76.8	1.424	0.238
Diastolic blood pressure	Time	7.5	1	7.5	0.826	0.367
	Group	1.2	1	1.2	0.13	0.719
	Time^*^Group	19.2	1	19.2	2.116	0.151
Irritability index	Time	0.074	1	0.074	0.01	0.922
	Group	46.875	1	46.875	2.485	0.12
	Time^*^Group	0.672	1	0.672	0.088	0.768
Stress index	Time	0.678	1	0.678	0.311	0.579
	Group	21.658	1	21.658	2.74	0.103
	Time^*^Group	1.87	1	1.87	0.856	0.359
Anxiety index	Time	132.32	1	132.32	14.4	0.000^**^
	Group	29.99	1	29.99	1.262	0.266
	Time^*^Group	7.505	1	7.505	0.82	0.369
Self-confidence index	Time	6.084	1	6.084	0.756	0.388
	Group	9.075	1	9.075	0.335	0.565
	Time^*^Group	0.672	1	0.672	0.083	0.774
Vitality index	Time	27.104	1	27.104	6.323	0.015^*^
	Group	46.888	1	46.888	4.696	0.034^*^
	Time^*^Group	12.656	1	12.656	2.952	0.091
Inhibition index	Time	27.075	1	27.075	17.371	0.000^**^
	Group	21.692	1	21.692	7.534	0.008^**^
	Time^*^Group	21.675	1	21.675	13.906	0.000^**^
Neuroticism index	Time	6.089	1	6.089	3.253	0.076
	Group	0.074	1	0.074	0.035	0.853
	Time^*^Group	6.665	1	6.665	1.958	0.167
Depression index	Time	2.691	1	2.691	0.714	0.402
	Group	14.707	1	14.707	4.348	0.051
	Time^*^Group	0.303	1	0.303	0.08	0.778
Pleasure index	Time	10.794	1	10.794	1.296	0.26
	Group	7.495	1	7.495	1.101	0.299
	Time^*^Group	2.697	1	2.697	0.324	0.571
Composure	Time	16.853	1	16.853	3.976	0.052
	Group	72.091	1	72.091	3.537	0.065
	Time^*^Group	9.902	1	9.902	2.145	0.148
Personality orientation	Time	3.335	1	3.335	0.01	0.956
	Group	67.47	1	67.47	3.589	0.061
	Time^*^Group	24.282	1	24.282	2.964	0.09
Self-regulation	Time	21.684	1	21.684	3.448	0.068
	Group	16.883	1	16.883	1.002	0.321
	Time^*^Group	0.075	1	0.075	0.12	0.914
Communication and expression skills	Time	0.302	1	0.302	0.016	0.899
	Group	0.298	1	0.298	0.014	0.908
	Time^*^Group	24.372	1	24.372	1.304	0.258
Reaction speed	Time	97.2	1	97.2	9.685	0.003^**^
	Group	120	1	120	7.8	0.007^**^
	Time^*^Group	24.246	1	24.246	2.416	0.126
Stress Tolerance	Time	36.322	1	36.322	2.382	0.128
	Group	30.04	1	30.04	1.794	0.186
	Time^*^Group	50.648	1	50.648	3.322	0.794
Learning adaptability	Time	0.074	1	0.074	0.005	0.942
	Group	46.925	1	46.925	3.396	0.07
	Time^*^Group	0.079	1	0.079	0.004	0.948
Self-management skills	Time	0.304	1	0.304	0.008	0.931
	Group	43.224	1	43.224	1.013	0.318
	Time^*^Group	19.184	1	19.184	0.477	0.492
Risk tolerance	Time	0.298	1	0.298	0.016	0.899
	Group	1.204	1	1.204	0.048	0.828
	Time^*^Group	1.208	1	1.208	0.066	0.798
Innovation capabilities	Time	10.812	1	10.812	0.386	0.537
	Group	58.8	1	58.8	1.791	0.186
	Time^*^Group	4.832	1	4.832	0.173	0.679
Proactive execution	Time	27.085	1	27.085	1.343	0.251
	Group	9.081	1	9.081	0.403	0.528
	Time^*^Group	6.053	1	6.053	0.3	0.586
Crisis management skills	Time	24.237	1	24.237	1.941	0.169
	Group	19.176	1	19.176	1.317	0.256
	Time^*^Group	29.97	1	29.97	2.4	0.127
RT(2-back)	Time	136668.6	1	136668.6	103.6	0.000^**^
	Group	94432.009	1	94432.009	52.897	0.000^**^
	Time^*^Group	13553.6	1	13553.6	10.281	0.002^**^
ACC(2-back)	Time	0.011	1	0.011	5.957	0.018^*^
	Group	8.33	1	8.33	0.01	0.982
	Time^*^Group	0.003	1	0.003	1.852	0.179
RT(Go/No-Go)	Time	38913.12	1	38913.12	27.973	0.000^**^
	Group	4431.835	1	4431.835	3.479	0.067
	Time^*^Group	9724.681	1	9724.681	6.991	0.011^*^
ACC(Go/No-Go)	Time	0.003	1	0.003	0.515	0.476
	Group	0.003	1	0.003	0.541	0.465
	Time^*^Group	0.012	1	0.012	2.491	0.12

^“*^″ indicates that *P* < 0.05 has a significant difference; ^“**^″ indicates a highly significant difference with *P* < 0.01.

As shown in Table 5, results of a simple effects analysis showed that the inhibition index exhibited a significant difference only in the experimental group (*P* < 0.01), whilst no significant change was observed in the control group. With regard to cognitive performance, both the control and experimental groups demonstrated a marked reduction in reaction time on the 2-back task (*P* < 0.01). However, the extent of improvement was greater in the experimental group. In contrast, reaction times on the Go/No-Go task were significantly reduced in the experimental group (*P* < 0.01), whilst no significant change was observed in the control group. These results indicate that the FOOTIBKE intervention effectively enhanced inhibitory control and executive function.

**Table 5:** Results of simple effects analysis.

Indicators	Group	Comparison	Mean Difference	95% confidence interval of the difference	*P*
Inhibition index	Control group	Pre vs. Post	0.1	−0.545–0.745	0.758
	Experimental group	Pre vs. Post	1.8	1.155–2.445	0.000^**^
RT(2-back)	Control group	Pre vs. Post	46.24	27.474–65.006	0.000^**^
	Experimental group	Pre vs. Post	88.751	69.985–107.516	0.000^**^
RT(Go/No-Go)	Control group	Pre vs. Post	18.011	−1.266–37.288	0.066
	Experimental group	Pre vs. Post	54.02	34.743–73.297	0.000^**^

^“**^″ indicates a highly significant difference with *P* < 0.01.

## 4 Discussion

The present study demonstrated that FOOTBIKE exercise significantly enhanced mental health in university students, particularly in domains related to emotional regulation, executive function, and stress perception, and cognitive responsiveness. Notably, although no substantial changes were observed in basic physiological indicators following the 8-week intervention, significant improvements were identified in psychological and executive-function-related measures. These findings suggest that the beneficial effects of FOOTBIKE exercise may be primarily associated with enhanced cognitive control and stress adaptation mechanisms rather than general physiological changes.

This study found that the observed reductions in anxiety and inhibition, as well as the increase in stress tolerance, are broadly consistent with previous evidence suggesting that regular physical activity can enhance psychological resilience (Connor and Davidson, 2003). However, our findings also suggest that the psychological effects of FOOTBIKE may differ from those reported for traditional aerobic exercise. Unlike interventions that emphasize exercise intensity or energy expenditure, FOOTBIKE requires constant adjustment of posture, balance, movement rhythm and environmental awareness. These additional cognitive demands may strengthen adaptive coping through repeated engagement in self-regulatory processes during exercise, thereby providing benefits beyond those expected from aerobic conditioning alone.

One notable finding was that improvements in executive function occurred alongside improvements in mental health. Although previous studies have consistently reported that exercise enhances executive function (Diamond, 2013; Esmaeilzadeh and Moradikor, 2025), the causal relationship between executive function and psychological well-being remains under debate. Some researchers have argued that cognitive improvements are secondary consequences of enhanced physical fitness, whereas others propose that executive function directly contributes to emotional regulation by strengthening top-down cognitive control. Our findings lend greater support to the latter interpretation because significant improvements were observed in executive-function-related measures despite minimal changes in conventional physiological indicators. This pattern suggests that enhanced cognitive control, rather than general physiological adaptation, may represent an important pathway through which FOOTBIKE promotes mental health.

The current findings are also consistent with previous studies demonstrating that moderate-intensity rhythmic exercise can improve cognitive control efficiency and reaction speed (Dkaidek et al., 2023). However, the magnitude of improvement observed in the present study may reflect characteristics unique to FOOTBIKE exercise. Unlike cycling or treadmill walking, FOOTBIKE requires simultaneous motor planning, dynamic balance control, environmental monitoring, and rapid movement adjustment. These concurrent cognitive and motor demands are consistent with the concept of cognitively engaging physical activity proposed by (Mao et al., 2024). Therefore, it is plausible that the superior psychological outcomes observed in the present study result from the integration of cognitive and motor challenges rather than from rhythmic movement alone.

Another noteworthy finding was that improvements in mental health occurred despite minimal changes in conventional physiological indicators such as heart rate, respiratory rate, and heart rate variability. Previous exercise studies often attribute psychological benefits to improvements in cardiovascular fitness or autonomic regulation (Albini et al., 2025). However, our findings suggest that these physiological adaptations may not be necessary prerequisites for short-term improvements in mental health. One possible explanation is that the 8-week intervention was sufficient to induce cognitive and emotional adaptations but insufficient to elicit detectable changes in resting physiological measures. Alternatively, conventional physiological indices such as resting heart rate and blood pressure may be less sensitive than neurocognitive measures for capturing early adaptations associated with cognitively engaging exercise.

An additional contribution of the present study lies in the integration of machine learning with multidimensional psychological assessment. Rather than relying solely on conventional statistical comparisons, the random forest model identified reaction speed, inhibitory control, and stress tolerance as the variables that most strongly distinguished participants following the intervention. These findings provide convergent evidence supporting the hypothesis that executive-function-related processes are central to the psychological effects of FOOTBIKE exercise. Nevertheless, because machine learning identifies predictive rather than causal relationships, future studies combining neuroimaging or longitudinal mediation analyses will be necessary to verify whether executive function directly mediates exercise-induced improvements in mental health.

Several limitations should be acknowledged. First, the relatively small sample size may limit the generalizability of the findings, and future studies should recruit larger multi-center samples to improve external validity. Second, although the present study incorporated AI-based psychological and physiological assessment systems together with machine learning analyses, future research should further integrate multimodal neurophysiological measurements, such as electroencephalography (EEG), electro dermal activity (EDA), and functional near-infrared spectroscopy (fNIRS), to better clarify the neural mechanisms underlying the observed cognitive and psychological improvements. Third, the current intervention duration was limited to 8 weeks. Future studies should explore the long-term effects of FOOTBIKE exercise and compare the differential effects of varying exercise intensities and intervention durations in order to establish more precise exercise prescriptions for mental health enhancement. Finally, future FOOTIKE interventions could go beyond individual-oriented exercises to incorporate structured interpersonal and collaborative components. For example, pupils could take part in FOOTIKE activities in pairs or groups, involving collaborative tasks, communication, coordinated movement, joint decision-making and problem-solving. This approach could create more opportunities for peer interaction and social engagement during exercise, and may extend the benefits of FOOTIKE from individual cognitive and emotional functioning to broader educational outcomes.

The findings of this study have important practical implications for promoting mental health among university students. Despite minimal changes in traditional physiological indicators, FOOTBIKE significantly improved psychological and executive-function-related outcomes, suggesting that cognitively engaging physical activity may provide an alternative approach to mental health promotion. This may be particularly relevant for students experiencing psychological stress or difficulties in emotional and cognitive regulation. FOOTBIKE combines motor planning, dynamic balance, rhythmic movement, environmental monitoring, and rapid movement adjustment, thereby integrating cognitive and motor demands. These characteristics support its potential application in university physical education, student health programs, and preventive mental health interventions. Moreover, improvements in executive function, cognitive responsiveness, emotional regulation, and stress tolerance, together with the random forest findings identifying reaction speed, inhibitory control, and stress tolerance as important variables, suggest that future exercise programs should consider not only frequency, duration, and intensity, but also the cognitive and psychological demands of exercise. Therefore, FOOTBIKE may serve as a complementary, function-oriented strategy for integrating physical activity and mental health promotion in higher education.

## 5 Conclusion

In conclusion, this study provides evidence that an 8-week FOOTBIKE exercise intervention can improve the mental health of university students. In addition to demonstrating overall psychological benefits, these improvements are closely associated with enhancements in executive function-related abilities, highlighting the potential role of cognitive engagement in physical activity in supporting mental health. The combination of multidimensional psychological assessment and machine learning analysis further identified reaction time, inhibitory control and stress tolerance as key indicators associated with improvements in mental health. These findings provide new evidence that processes related to executive function may be an important pathway through which cognitive engagement in physical activity promotes mental health, whilst also illustrating the value of combining artificial intelligence technologies with psychological assessment to better understand exercise-induced changes in mental health. Given that FOOTBIKE was associated with improvements in executive function, emotional regulation, cognitive responsiveness, and stress tolerance, intervention activities could include rapid decision-making, response inhibition, motor planning, dynamic balance, environmental monitoring, and progressively challenging movement tasks. These objectives should be assessed using relevant cognitive and psychological indicators, such as reaction speed, inhibitory control, emotional regulation, and stress tolerance, to enable individualized adjustment of intervention content. Accordingly, integrating structured FOOTBIKE training with brief cognitive or psychological tasks may help combine physical education with mental health promotion and foster cognitive-emotional skills relevant to students' academic performance and psychological well-being.

## Data Availability

The raw data supporting the conclusions of this article will be made available by the authors, without undue reservation.
