# Supplementary material for: FOOTBIKE exercise enhances mental health through improved executive function in university students
Source: Front Psychol. 2026 Oct 26;17:1891734. doi: 10.3389/fpsyg.2026.1891734 (PMC13745347; doi:10.3389/fpsyg.2026.1891734)
Supplement: Supplementary file 1 [file Table_1.docx]

**Supplementary Materials**

**Text S1. The AI Physiological and Mental Health Assessment System**

The AI Physiological and Mental Health Assessment System, developed by Shenzhen Technology (Nanjing) Co., Ltd., a subsidiary of China Today Education Group, utilizes video image micro-vibration analysis technology combined with cutting-edge optical blood flow imaging technology. It is a technologically advanced system for assessing mental and physiological health, characterized by a high degree of innovation. The system offers rapid assessment and a comprehensive range of indicators, providing a vital basis for the early identification of mental health issues and their timely resolution, thereby securing a valuable window of opportunity. The system has been certified by experts, as shown in Figure S1 and S2.

The principle behind AI-based mental and physical health monitoring systems: by selecting the pixel coordinates of a specific area of the face, recording changes in greyscale values over time, and analyzing the time series of images, the system analyses a person’s emotional state. For example, when a person feels tense or anxious, their facial muscles tense up, causing the image to vibrate slightly; when a person feels happy and relaxed, their facial muscles relax, resulting in only a faint vibration in the image (Baker et al., 2025). An example of the system in action is shown in Figure S3.

Examples of algorithms for AI-based mental and physical health monitoring systems: x, y – the coordinates of a point; Ux, y, i – the amplitude of the signal at point x, y in frame i; Ux, y(i+1) – the amplitude of the signal at point x in frame (i+1); N – the cumulative number of frames in which an amplitude component of magnitude a is present.

The display of the movement frequency at each point on the object (effectively in space) produces an image similar to one with an amplitude of 1, but when displayed using a colour scale, it differs slightly from the amplitude; regardless of the amplitude, a change in the signal at a point is recorded, so the value is either 0 or 1.

The relative frequency component at each point of vibration is determined by the following equation:

$$F_{x,y}=\frac{F_{m}}{N}\sum_{i=1}^{N} \left\{ \begin{aligned} 1,\Delta_{i}\neq0 \\ 0,\mathrm{otherwise} \end{aligned} \right.$$

where $\Delta_{i}$is the inter-frame difference at the first point of the image; *N* is the cumulative number of frames in which the amplitude component a is present. In order to convert the relative frequency into an actual physical frequency with the unit Hz, it is necessary to adjust the obtained value to correspond to the number of frames processed per second. As it is not possible to display two numbers simultaneously at a single point on a two-dimensional image, the amplitude is observed visually, whilst the frequency image of the object is displayed sequentially on the monitor screen. The given formula confirms the actual amplitude, which represents the magnitude of the signal variation at each spatial point, whilst the actual frequency of the micro-vibrations in the video image represents the frequency of the signal variation at that spatial point (Zhu et al., 2026).

The indicators in the AI-based physiological and mental health monitoring system—including the Irritability Index, Stress Index, Anxiety Index, Inhibition Index, Reaction Capacity and Stress Tolerance—are inferential indicators derived through comprehensive calculations based on physiological signals, behavioral performance and AI algorithm models. This assessment system utilizes multimodal data—including heart rate, heart rate variability, skin conductance response, respiratory rate and performance on cognitive tasks—to calculate comprehensive indicators such as the Irritability Index, Stress Index, Anxiety Index, Inhibition Index, Reaction Capacity and Stress Tolerance via machine learning algorithms. Specifically, the Irritability Index, Stress Index and Anxiety Index primarily reflect emotional arousal and autonomic nervous system stress levels; the Inhibition Index reflects inhibitory control within executive function; Reaction Capacity reflects the speed and accuracy of cognitive processing; and Stress Tolerance reflects an individual’s physiological regulation and recovery capacity under stressful conditions.

Reference

Baker, J., Ngo, H., Efthimiou, T. N., Elsenaar, A., Mehu, M., and Korb, S. (2025). Electrical stimulation of smiling muscles reduces visual processing load and enhances happiness perception in neutral faces. *Commun Psychol, 3*(1), 94. doi:10.1038/s44271-025-00281-y

**Figure S1. Expert opinions on AI Physiological and Mental Health Assessment System, Chinese stamped version**

**Figure S2. Expert opinions on AI Physiological and Mental Health Assessment System in English**

**Figure S3. Real-life examples**

**Figure S4. The spearman’s relations among all the variables**

**Table S1. Daily schedule of control group college students**

| Time  weekday | 8:00-9:30 | 10:00-11:30 | 13:30-15:00 | 15:30-17:00 |
| --- | --- | --- | --- | --- |
| Monday | College Chinese and Applied Writing | Sports Anatomy | - | Fundamentals of Traditional Chinese Medicine |
| Tuesday | - | Pedagogy | College Physical Education (Athletics) | - |
| Wednesday | College Chinese and Applied Writing | Ideology, Morality, and Rule of Law | College Foreign Language | Sports Anatomy |
| Thursday | - | College Physical Education (Football) | Introduction to Sports | - |
| Friday | Sports Anatomy | Ideology, Morality, and Rule of Law | Fundamentals of Innovation and Entrepreneurship | - |
